# Apoptotic effect of a novel kefir product, PFT, on multidrug-resistant myeloid leukemia cells via a hole-piercing mechanism

**DOI:** 10.3892/ijo.2014.2258

**Published:** 2014-01-15

**Authors:** MAMDOOH GHONEUM, JAMES GIMZEWSKI

**Affiliations:** 1 Department of Otolaryngology, Charles R. Drew University of Medicine and Science, Los Angeles, CA 90059;; 2 Department of Chemistry and Biochemistry, University of California, Los Angeles (UCLA), California Nanosystems Institute at UCLA, Los Angeles, CA 90095, USA

**Keywords:** *Lactobacillus kefiri*, apoptosis, HL60/AR cells, caspase

## Abstract

We examined the apoptotic effect of a novel Probiotics Fermentation Technology (PFT) kefir grain product; PFT is a natural mixture composed primarily of *Lactobacillus kefiri* P-IF, a specific strain of *L. kefiri* with unique growth characteristics. The aim of this study was to examine the apoptotic effect of PFT on human multidrug-resistant (MDR) myeloid leukemia (HL60/AR) cells *in vitro* and explore the mechanistic approach underlying its effect. HL60/AR cells were cultured with PFT (0.6–5.0 mg/ml) for 3 days. The apoptotic effect of PFT was assessed through examination of percent apoptosis, caspase 3 activation, Bcl-2 expression levels and changes in mitochondrial membrane potential (MMP). PFT induced apoptosis in HL60/AR cells in a dose-dependent manner which was maximal at 67.5% for 5 mg/ml. Induction of apoptosis was associated with activation of caspase 3, decreased expression of Bcl-2 and decreased polarization of MMP. In addition, PFT showed a unique characteristic of piercing holes in HL60/AR cells, as indicated by AFM studies. This hole induction may be responsible for the apoptotic effect on cancer cells. These results suggest that PFT may act as a potential therapy for the treatment of MDR leukemia.

## Introduction

Lactic acid bacteria (LAB) have been used throughout history to produce fermented food and milk products. In the early 1900s, biologist Eli Metchinkoff suggested that the intake of LAB could increase the life span of humans (1). His findings have been verified by many scientists who have found that ingesting LAB fermented products provides many benefits for maintaining health (2,3). LAB strains present in fermented milk are normal components of intestinal microflora and help maintain a healthy balance of probiotic bacteria while reducing pathogenic bacteria (4,5). In addition to gut maintenance, LAB have more recently been tested to treat a variety of diseases including Crohn’s disease, rheumatoid arthritis and cancer (6).

The idea of treating cancers with LAB is not as recent as one might think. Over a century ago, Metchnikoff suggested that LAB had a protective effect against colorectal cancer (7). However, it is the recent resurgence of research concerning the microbiotic influence on human health that has spurred researchers to confirm Metchnikoff’s findings on the anti-cancer ability of LAB in animal models and to expand understanding of LAB’s influence on the cellular environment (8–12). Additional research has shown that LAB also exerts anticancer activity against other types of cancer such as breast and ovarian cancer (13–17).

Conventional treatments for cancer such as chemotherapy aim to initiate apoptosis. However, these drugs can be toxic and can decrease a cancer patient’s quality of life. Therefore, there has been an effort to investigate alternative treatments that have fewer side effects and improve the health of the patient. A recent study has shown that *Lactobacillus reuteri* enhanced tumor necrosis factor (TNF)-induced apoptosis in human chronic myeloid leukemia-derived cells (18) and that bacterial soluble factors secreted by *Lactobacillus casei rhamnosus* caused induction of apoptosis in human monocytic leukemia-cell line, THP-1 (19). These data suggest that fermented milk products and/or the fermentative bacteria themselves may have chemoprotective effects without the toxic side effects of conventional therapeutic drugs.

The product we use in this study is a symbiotic microbe of lactic acid bacteria and yeasts known as Probiotics Fermentation Technology (PFT) kefir grain product. PFT is separated from kefir, a popular drink across Eastern and Northern Europe and Russia. Kefir has been shown to have various health benefits, for example, it protects the intestine against disease-causing bacteria (20) and its kefiran component was shown to lower high blood pressure and reduce serum cholesterol levels in rats (21). PFT mainly contains a unique *Lactobacillus kefiri* P-IF (*L. kefiri* P-IF) strain that has a unique DNA sequence and shows a 99.6% homology with regular kefiries and similar 16S ribosome sequence compared with other *L. kefiri* strains. Although strain P-IF shares many common characteristics with other *L. kefiri* strains, P-IF also has many distinct features that may contribute to its effectiveness as an anticancer agent (Table I) (22). Unlike other *L. kefiri* strains, P-IF utilizes galactose as a carbon source and produces carbonic acid when its growth medium is agitated. Most *L. kefiri* strains grow in a lengthwise-dimensional pattern, however, P-IF grows three-dimensionally, which is attributed to the unique carbohydrate chains on its surface (22).

The results of this study show that PFT possesses the ability to pierce holes in MDR human myeloid leukemic (HL60/AR) cells, which induces apoptosis in the cancer cells by intrinsic (mitochondrial) pathway of apoptosis. This study suggests that PFT may exert a therapeutic effect in treating MDR cancers.

## Materials and methods

### Tumor cell line and culture conditions

Human multidrug-resistant (MDR) myeloid leukemia (HL60/AR) cells were used in the present study. Cells were kindly provided by Dr S. Gollapudi at the University of California, Irvine, CA, USA. Tumor cells were maintained in our laboratory in a complete medium (CM) that consisted of RPMI-1640, 10% fetal calf serum (FCS), 2 mM glutamine and 100 *μ*g/ml streptomycin and penicillin.

### Drugs and chemicals

3-[4,5-dimethylthiazol-2-yl]-2,5-diphenyltetrazolium bromide (MTT) (Sigma-Aldrich, St. Louis, MO, USA), was employed.

### Probiotics Fermentation Technology (PFT) kefir grain product

**Figure 1.**
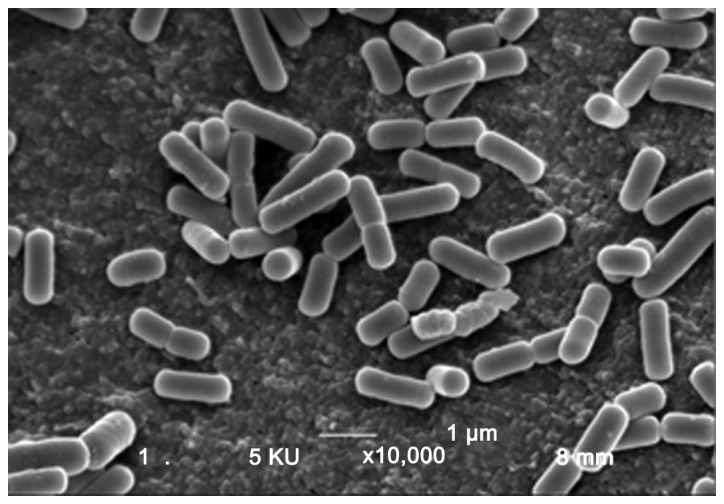
*L. kefiri* P-IF can grow in three dimensions. Electron microscope image of *L. kefiri* P-IF strain. *L. kefiri* P-IF are rod-shaped bacteria that uniquely adhere to one another in all three dimensions.

PFT is a mixture that mainly (∼90%) contains a freeze-dried form of heat-killed *L. kefiri* P-IF; it is a specific strain of LAB that has a unique DNA sequence and PET scans show a 99.6% homology with regular kefiries. Characteristics of *L. kefiri* P-IF are shown in Table I and Fig. 1. PFT also contains ∼2–3% each of bacterial strain, *Lactobacillus kefiri* P-IF and *Lactobacillus kefiri* P-B1 and three yeast strains, *Kazachstania turicensis, Kazachstania unispora* and *Kluyveromyces marxianus* (22). PFT was provided by Paitos Co., Ltd. Yokohama, Kanagawa, Japan.

### Detection of cancer cell viability using propidium iodide

HL60/AR cells were cultured in the presence or absence of PFT at different concentrations (0, 0.6, 1.25, 2.5 and 5 mg/ml) for 3 days and the percentage of dead cancer cells was examined by the propidium iodide (PI) technique using a FACScan flow cytometery. Briefly, PI was added to the cells (1×10^6^/ml) to give a final PI concentration of 50 *μ*g/ml. The cells were stained for 30 min at room temperature in the dark and analyzed by FACScan (Becton-Dickinson, San Jose, CA, USA).

### Expression of Bcl-2

For detection of Bcl-2, cells were first fixed and permeabilized with ice-cold 70% methanol. Cells were then stained with FITC-labeled anti-Bcl-2 or isotype control (Dako Corp., Carpinteria, CA, USA), washed and analyzed by FACScan. The percentage of cells expressing Bcl-2 and mean fluorescent intensity (an indicator of density of the molecules/cell) was determined.

### Intracellular activity of caspase 3

The method for measuring intracellular activity of caspase 3 is based on carboxyfluorescein labeled fluromethyl ketone (FMK)-peptide inhibitors of caspases. These inhibitors are cell permeable and non-toxic. Once inside the cells, these inhibitors bind covalently to the active caspase. Caspase-positive (+) cells are distinguished from caspase-negative (−) cells with the aid of flow cytometry. Briefly, cells undergoing apoptosis were loaded with fluorescein labeled FAM-DEVD-FMK for caspase 3 (Intergen Co., NY, USA). After 1-h incubation, the cells were washed to remove unbound caspase and cells that contained bound inhibitor were quantified using a FACScan flow cytometer.

### Detection of mitochondrial membrane potential (MMP)

Variations of the mitochondrial transmembrane potential ΔΨm during apoptosis were studied using tetramethylrhodamine ethylester (TMRE, Molecular Probes, Eugene, OR, USA). Briefly, after treatment with PFT for 3 days, cancer cells (5×10^5^ cells/ml) were incubated with 50 nM TMRE for 30 min at 37°C. The cells were washed with PBS and analyzed with FACS Forward, the side scatters were used to gate and exclude cellular debris using a FACScan. The cells were excited at 488 nm and the emission was collected on the FL2 channel. Five thousand cells were analyzed. The data were acquired and analyzed using CellQuest software (Becton-Dickinson).

### AFM imaging

HL60/AR cells (1×10^6^ cells/ml) were cultured with PFT (5 mg/ml) for 2 min and 24 h. Results were compared to those of cells without treatment. Cytospin preparations (Shandon Southern Inst., Sewickley, PA, USA) of cells were air-dried, fixed in 100% MeOH for 5 min and prepared for AFM studies. AFM studies were carried out to examine the morphological changes associated with PFT treatment of HL60/AR cells such as hole induction and membrane blebbing. Dimension 5000 AFM (Veeco) under contact mode was used to image the HL60/AR cells with Bruker’s Sharp Nitride Lever (SNL) silicon probes (Veeco). Topographic height images were recorded at 512×512 pixels at a scan rate of 0.8 Hz. Image processing was performed using SPIP^TM^ Software. Usually an MLCT-AFM tip (with a ‘k’ value of 0.03N/m) contributes to the broadening effect because of its specific geometry (23).

### Statistical analysis

**Figure 2.**
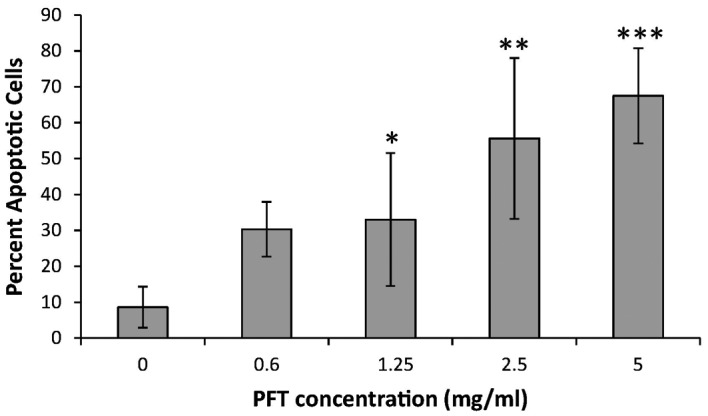
PFT induces apoptosis of HL60/AR cells. Tumor cells were cultured with PFT at different concentrations (0, 0.6, 1.25, 2.5 and 5 mg/ml) and the percentage of dead cancer cells was examined by the propidium iodide (PI) technique using a FACScan flow cytometry. Data represent the mean ± SD of 4 experiments. Statistical analysis compared the concentrations to control (0 mg/ml). ^*^p<0.05, ^**^p<0.0005, ^***^p<0.0001.

Statistical significance for cell apoptosis in Fig. 2 was determined by Student’s t-test. Differences were considered significant at the p<0.05 level. Statistical analysis for flow cytometry was performed by the Kolmogorov-Smirnov test using CellQuest Software system. A D-value of >0.2 was considered statistically significant.

## Results

### Effect of PFT on tumor cell survival

We examined the effect of PFT on tumor cell survival using propidium iodide and FACScan flow cytometry. The data in Fig. 2 demonstrate that treatment with PFT increased apoptosis in the cancer cells in a dose-dependent manner. Even at a lower concentration (0.6 mg/ml), the PFT induced apoptosis in ∼30% of the HL60/AR cells. The percentage of apoptosis continued to increase in conjunction with higher concentrations of PFT reaching an average of 67.5% apoptosis at 5 mg/ml.

### PFT induces activation of caspase 3

**Figure 3.**
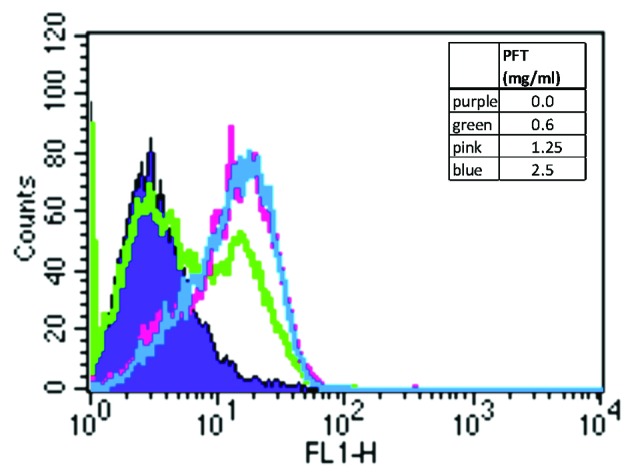
PFT induces apoptosis through activation of caspase 3. The activation of caspase 3 was determined in cancer cells post-culture with PFT at different concentrations (0, 0.6, 1.25 and 2.5 mg/ml). The lines represent cancer cells in the absence of PFT (control, purple) and cancer cells treated with PFT at concentration of 0.6 mg/ml (green), 1.25 mg/ml (pink), or 2.5 mg/ml (blue). The activation of caspase 3 in cells was detected using carboxyfluorescein-labeled cell permeable peptide substrate and was analyzed using flow cytometry.

To verify activation of the apoptotic pathway, the level of caspase 3 activation was investigated by treating HL60/AR cells with PFT at varying concentrations for 24 h and analyzed using flow cytometry. Data depicted in Fig. 3 show that the proportion of cancer cells with increased active caspase 3 was higher in PFT-treated cells than in control untreated cells. This would suggest that pre-exposure of HL60/AR cells to PFT led to increased activation of the executioner caspase 3.

### PFT depolarizes mitochondrial membrane potential (MMP)

**Figure 4.**
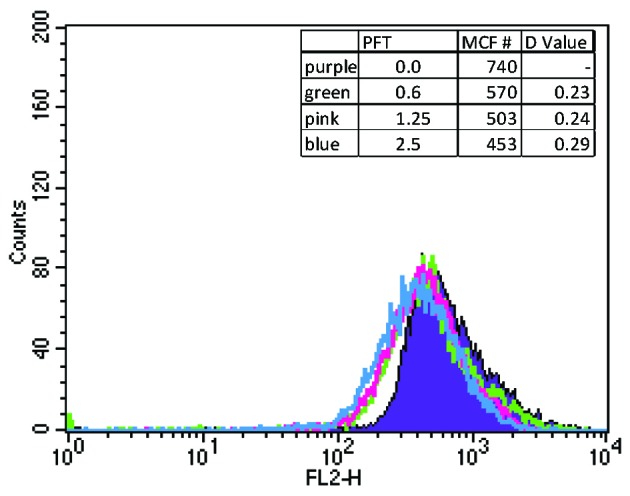
PFT reduces MMP. Tumor cells were cultured with PFT at different concentrations (0, 0.6, 1.25 and 2.5 mg/ml) and the MMP was determined by flow cytometry using TMRE dye. Decrease in TMRE fluorescence indicates loss of membrane potential. PFT depolarized membrane potential in a dose-dependent manner. Statistical analysis was performed by the Kolmogorov-Smirnov test using CellQuest Software System. A D-value >0.2 is considered statistically significant.

Experiments were carried out to examine the ability of PFT to disrupt MMP. HL60/AR cells were treated in the presence or absence of PFT at varying concentrations and MMP was determined by flow cytometry using membrane potential sensitive TMRE dye. The data in Fig. 4 show that treatment of cells with PFT resulted in a significant decrease in the mitochondrial polarization of cancer cells as compared to untreated cells.

### Expression of Bcl-2

**Figure 5.**
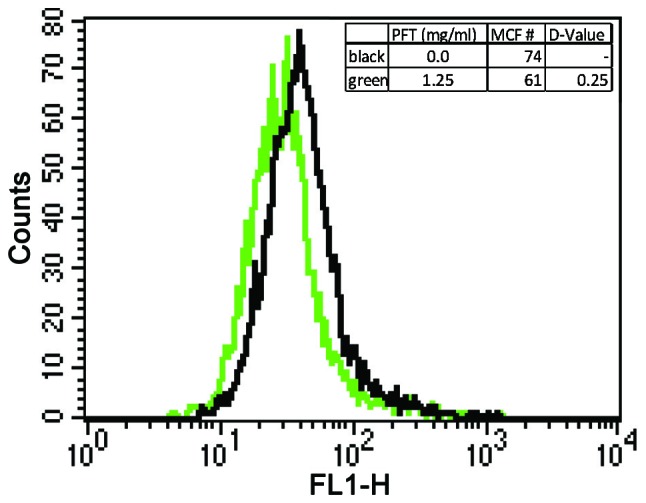
PFT reduces the expression of Bcl-2. HL60/AR cells (1×10^6^ cells/ml) were cultured with PFT at a concentration of 1.25 mg/ml for 24 h. Expression of Bcl-2 was determined by staining the cells with anti-human Bcl-2 antibody and by flow cytometry. It is shown as mean fluorescent intensity (MCF).

The expression of Bcl-2 was examined to determine the anti-apoptotic activity post-treatment of HL60/AR cells by PFT. Results depicted in Fig. 5 show that treatment with PFT caused a statistically significant decrease in expression of Bcl-2 compared to control cancer cells without treatment.

### AFM studies

**Figure 6.**
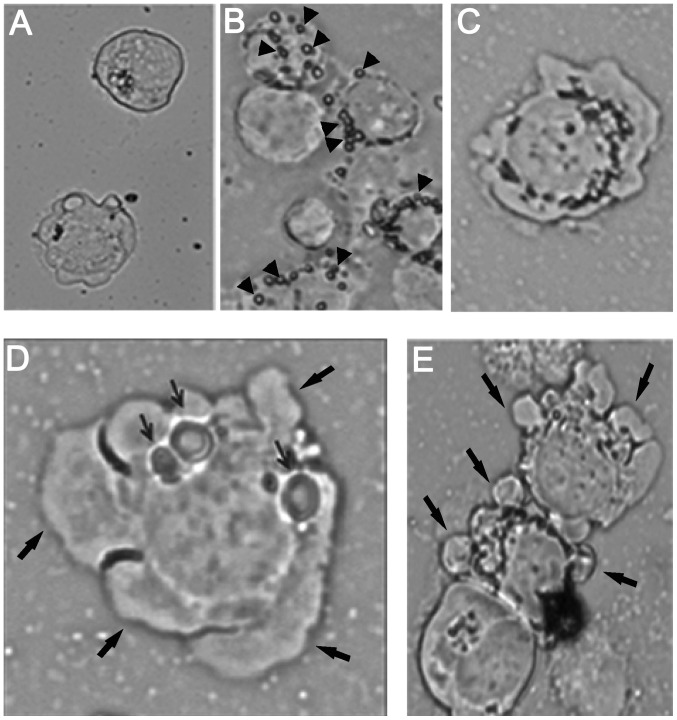
PFT induces holes and membrane blebbing in cancer cells. HL60/AR cells (1×10^6^ cells/ml) were cultured with PFT and the changes were examined by AFM. (A) Control untreated cells. (B) Cells treated with 5 mg/ml of PFT for 2 min; note the attachment of cancer cells to *L kefiri* P-IF (arrowheads). (C–E) Cells treated with 5 mg/ml of PFT for 24 h; note the presence of extensive holes surrounding the nucleus. (D) A cancer cell with three large holes (thin arrows). (D and E) Membrane blebbing of cancer cells is indicated by thick arrows. Cells were examined using optical imaging.

**Figure 7.**
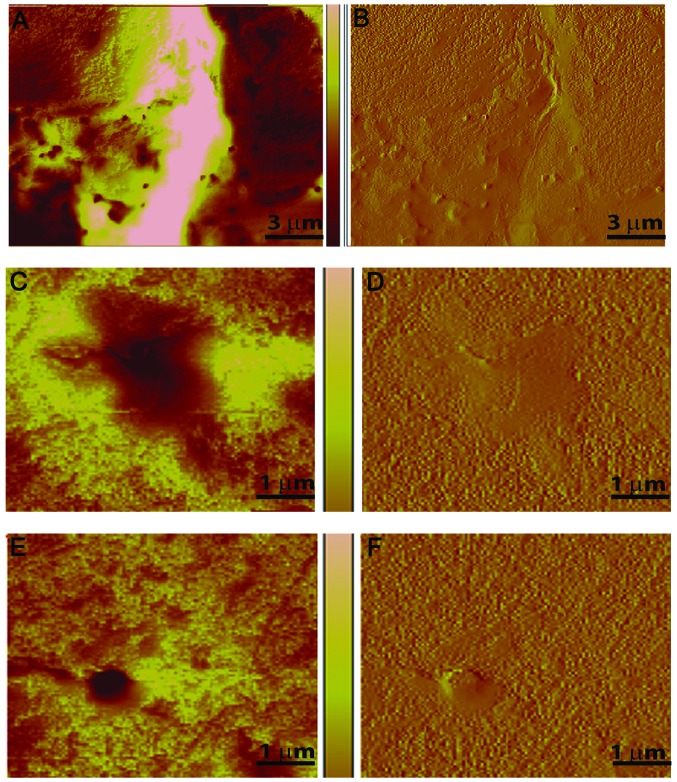
Hole detection using AFM (peak force imaging). Darker color indicates greater hole depth. Note the variation in hole shape, size and depth. (A and B) Several smaller, distinctly circular holes. (C and D) A broad, irregularly shaped, deep indentation. (E and F) A large, circular hole.

**Figure 8.**
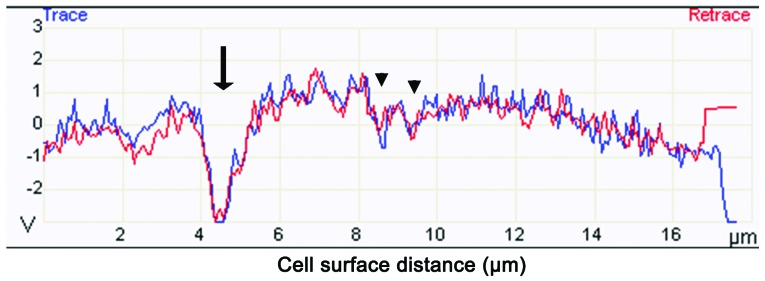
Determining the depth of PFT-induced hole formation. The red and blue lines indicate the surface contour of an HL60/AR cell treated with PFT. The arrow indicates a large hole detected by the SNL tip and arrowheads indicate smaller holes. This image is representative of many HL60/AR cells during PFT treatment.

AFM studies were carried out to examine the morphological changes associated with PFT treatment of HL60/AR cells. Results showed a low percentage of the control untreated HL60/AR cells with holes, as well as a small number of holes per cell (Fig. 6A). When the cancer cells were exposed to *L. kefiri* P-IF (5 mg/ml) for 2 min, we observed an adherence of *L. kefiri* P-IF to the cancer cells (Fig. 6B). Exposure of cancer cells to *L. kefiri* P-IF for a longer time (24 h) resulted in 2.6-fold increase in the percentage of cancer cells with holes. Control untreated cells showed 17.5% had single holes and 2% had multiple holes. However, of the *L. kefiri* P-IF-treated cells, 42% had single holes and 9.5% had multiple holes. Fig. 6C shows multiple holes surrounding the nucleus. Some of the holes are highly enlarged, as can be seen in Fig. 6D. The holes were situated in the cytoplasm and the nucleus. Fig. 6D and E also shows that hole induction was associated with membrane blebbing and a decrease in the nuclear to cytoplasmic ratio. These morphological changes are considered to be signs of cancer cell apoptosis. The holes were further characterized by peak force imaging (Figs. 7 and 8), where the variation in size, shape and depth of the holes was measured. The color intensity indicates hole depth, with lighter areas being shallow or flat and darker colors indicating depth (Fig. 7). The topography of the cell was traced using a cantilever tip. The changes in the vertical movement of the SNL tip were recorded and visualized in graphic form as seen in Fig. 8. A decrease along the y-axis indicates the presence of a hole on the surface of the cell. During PFT treatment, many holes were detected in HL60/AR cells using this method of analysis (Fig. 8).

## Discussion

In this study, we examined the apoptotic activity of PFT, a mixture with the main constituent of *L. kefiri* P-IF. While yogurt consumption and exposure to LAB has been shown to exert an inhibitory effect on the growth of different types of cancers (9,11,14–17), no study has examined the apoptotic effects of LAB against MDR cancer cells nor the possible mechanism underlying its effect on these cells. Our results show that PFT has the ability to induce an apoptotic effect on MDR cancer cells in a dose-dependent manner, which is maximal at 67.5% at concentration of 5 mg/ml. This ability of PFT holds great potential because the development of MDR to chemotherapy by tumor cells presents great obstacles with the current treatment options for cancer. Several drugs have been developed as sensitizers for chemotherapeutics, such as the membrane transporter P-glycoprotein (P-gp) (24,25), 12-deoxyphorbol 13-phenylacetate (26), probenecid (27) and magnetic iron oxide nanoparticles (28,29) but these drugs are toxic.

Several studies have shown that LAB exerts antitumor activity via several mechanisms. These include the ability of LAB for binding mutagens and removing carcinogens from colon (30,31), modulation of different arms of the immune system such as NK cells, dendritic cells, B cells and T cells (32–34) and induction of apoptosis in cancer cells (18,35,36). Results of this study showed an interesting phenomenon whereby *L. kefiri* P-IF was able to pierce holes in HL60/AR cells. PFT induces apoptosis in HL60/AR cells associated with activation of caspase 3, decreased expression of Bcl-2 and decrease in the polarization of MMP. This suggests that these holes appear to be responsible for the induction of apoptosis of cancer cells. In addition, AFM studies show that cancer cells with holes are correlated with signs of apoptosis such as membrane blebbing and decrease in the nuclear to cytoplasmic ratio. The mechanism by which uptake of *L. kefiri* P-IF induces an apoptotic effect on cancer cells may involve [Ca^2+^]I. Our earlier studies showed that human breast cancer MDA-MB-231 cells underwent apoptosis following phagocytosis of heat-killed yeast *S. cerevisiae* (37) by a mechanism that involved the elevation of [Ca^2+^]I. This was evidenced by an increase of [Ca^2+^]I post-uptake of yeast and also in the inhibition of apoptosis post-addition of 2-aminoethoxydiphenyl borate (2APB), a pharmacological inhibitor of Ca^2+^ release from the endoplasmic reticulum. We believe that the uptake of *L. kefiri* P-IF by cancer cells may be responsible for the apoptotic effect by this agent.

Hole piercing has been observed with nanoparticles such as nickel (38) and DPV576, a mixture of nanodiamond and nanoplatinum in liquid (39). It is noteworthy that the hole piercing ability of DPV576 has been associated with reversing drug resistance in HL60/AR cancer cells by increasing the accumulation of chemotherapeautic drugs such as daunorubicin inside cancer cells (39). Therefore, we believe that hole induction by *L. kefiri* P-IF may likewise facilitate the uptake and accumulation of chemotherapeutic drugs by MDR cancer cells, in a manner similar to DPV576 nanoparticles. This is an area for future study.

Vacuoles appear in the cancer cells apparently due to the cells ability to phagocytize microorganisms, such as bacteria and yeast (40–43) and other cells, including: erythrocytes, lymphocytes and neutrophils (44–46). Furthermore, research has also revealed the cannibalistic ability of tumor cells to phagocytize other cancer cells (47). In the present study, we observed that cancer cells treated with *L. kefiri* P-IF revealed a 2.6-fold increase in the percentage of cells with holes as compared to control untreated HL60/AR cells. This increase was observed in both cancer cells with single as well as multiple holes, which were situated in the cytoplasm and in the nucleus.

The unique properties of the *L. kefiri* P-IF strain may account for its ability to induce apoptosis in the MDR HL60/AR leukemic cell line. The specific three-dimensional growth of the P-IF colony indicates that the surface proteoglycans differ from other *L. kefiri* strains (22). The cell surface of bacteria and other microorganisms are composed of varying polysaccharide-peptide complexes that have been shown to have specific stimulatory effects on host cells that can be beneficial or inhibitory for cell growth (48–50). Further investigation into the unique cell wall components and attributes of *L. kefiri* P-IF could contribute to a better understanding of the apoptotic induction by cell surface proteoglycans. While not investigated in this study, *L. kefiri* P-IF has the ability to survive at a low pH, indicating that the P-IF strain remains viable as it travels through the stomach into the intestine, allowing it to continue to grow and provide a protective effect. Some of the protective effects of the P-IF strain may come from its ability to metabolize galactose. High levels of galactose can cause toxicity which could lead to mutations and health problems (51). The presence of *L. kefiri* P-IF in the gut may help to prevent damaging levels of galactose (52). This property warrants future investigation for prevention and maintenance of general health.

*L. kefiri* P-IF has been shown to be a non-toxic agent. Results of earlier studies in mice treated with *L. kefiri* P-IF showed that no macroscopic or histopathological abnormalities were detected in different organs post-treatment and no changes in the body weight as compared with control untreated mice (53).

In conclusion, *L. kefiri* P-IF represents a novel symbiotic microbe culture that exerts an apoptotic effect on HL60/AR cells by a mechanism that may involve piercing holes in the cellular membrane of cancer cells. *L. kefiri* P-IF may represent a new class of adjuvants that could be used to improve the treatment of MDR leukemia.

## Figures and Tables

**Table I.** *L. kefiri* P-IF strain characteristics.

Test	P-IF characteristic
*Lactobacillis kefiri*	99.6% sequence homology
	16S ribosome identification
Cell shape	Rod
Gram staining	+
Motility	Non-motile
Colony growth	3D growth^a^
Carbon utilization	Glucose
	Fructose
	Galactose^a^
	L-arabinose
	Ribose
	Maltose
	Lactose
	Melibiose
	Gluconate
Acid/gas production	Carbonic acid gas^a^
pH tolerance	>4.3 pH^a^

a Characteristics not common in other kefiri strains.
